# Exercise-induced myokines downregulates the ACE2 level in bronchial epithelial cells: Implications for SARS-CoV-2 prevention

**DOI:** 10.1371/journal.pone.0271303

**Published:** 2022-07-20

**Authors:** Vaishali Bhardwaj, Mart Dela Cruz, Deepika Subramanyam, Rohit Kumar, Sandeep Markan, Beth Parker, Hemant K. Roy

**Affiliations:** 1 Department of Medicine, Baylor College of Medicine, Houston, Texas, United States of America; 2 Department of Anaesthesiology, Baylor College of Medicine, Houston, Texas, United States of America; Waseda University: Waseda Daigaku, JAPAN

## Abstract

**Background:**

The Covid-19 pandemic has emerged as the leading public health challenge of our time (20^th^ century). While vaccinations have finally blunted the death rate, concern has remained about more virulent forms highlighting the need for alternative approaches. Epidemiological studies indicate that physical activity has been shown to decrease the risk of infection of some respiratory viruses. Part of the salutary effects of exercise is believed to be through the elaboration of cytokines by contracting skeletal muscles (termed myokines). The objective of this study was to investigate whether exercise-induced myokines would mitigate the SARS-CoV-2 infectivity of the bronchial epithelium through modulating the SARS-CoV-2 Covid-19 receptor (angiotensin-converting enzyme 2 -ACE2) its priming enzyme, transmembrane serine protease 2 (TMPRSS2).

**Methods:**

We utilized a cell culture model of exercise to generate myokines by differentiating C2C12 cells into myotubules and inducing them to contract *via* low-frequency electric pulse stimulation. Condition media was concentrated via centrifugation and applied to human immortalized human bronchial epithelium cell line (6HBE14o) along with conditioned media from unstimulated myotubules as controls. Following exposure to myokines, the 16HBE14o cells were harvested and subjected to quantitative RT-PCR and Enzyme-Linked Immunosorbent Assay (ELISA) for assessment of mRNA and protein levels of ACE2 and TMPRSS2, respectively. Pilot proteomic data was performed with isotope barcoding and mass spectroscopy.

**Results:**

Quantitative Real-Time PCR of 16HBE14o with 48 h treated unstimulated vs. stimulated myokine treatment revealed a reduction of ACE2 and TMPRSS2 mRNA by 32% (p<2.69x10^-5^) and 41% (p<4.57x10^-5^), respectively. The high sensitivity of ELISAs showed downregulation of ACE2 and TMPRSS2 protein expression in 16HBE14o cells by 53% (p<0.01) and 32% (p<0.03) respectively with 48 h treated. For rigor, this work was replicated in the human lung cancer cell line A549, which mirrored the downregulation. Proteomic analysis showed dramatic alteration in myokine profile between contracted and uncontracted C2C12 tubules.

**Conclusions:**

The current study explores a novel approach of a modified exercise cell culture system and uses ACE2 and TMPRSS2 as a surrogate marker of SARS-CoV-2 infectivity. In conclusion, we demonstrated biological data supporting exercise’s protective effect against Covid-19. These further strengthen myokines’ beneficial role as potential therapeutic targets against SARS-CoV-2 and similar viruses albeit these preliminary cell culture studies will require future validation in animal models.

## Introduction

COVID-19 is a major public health challenge with hundreds of millions infected and tens of millions of fatalities across the globe. This infection is caused by a specific member of the Coronaviridae family, SARS-CoV-2 [1]. While most patients are minimally symptomatic, a significant proportion developed the fulminant disease [2]. Although numerous organs can be impacted by SARS-CoV-2 infection, most fatalities are related to pulmonary infections that lead to acute respiratory distress syndrome (ARDS) [3]. The risk factors for more severe SARS-CoV-2 infection include age, hypertension, obesity, race. Pharmacotherapy has been developed from repurposed agents including remdesivir, dexamethasone, tocilizumab, baracitinib, molnupiravir, Paxlovid drug, monoclonal antibodies, etc. [4–6] however, the efficacy of these pharmacological interventions has been modest. The most promising precautionary measures include vaccinations with mRNA or adenovirus vectors [7]. These have made a considerable impact in stanching the pandemic fatalities, but concerns remain given vaccine hesitancy, waning immunity/need for boosters, unavailability of vaccines in many nations, and the emergence of more virulent variants (delta and omicron). Thus, other approaches are urgently needed.

In this regard, one promising strategy seems to be interrupting coronavirus interaction with the epithelial cell surface, potentially leading to decreased morbidity and transmissibility [2]. The SARS-CoV-2 viral entry into the target cells is facilitated by binding of the viral spike protein [8–12] to the cell surface receptor entry receptor angiotensin-converting enzyme 2 (ACE2) as an entry receptor [8,10]. This lock and key interaction is facilitated by modifications of the spike and transmembrane protease serine 2 (TMPRSS2) for S protein priming [9–11]. Moreover, these proteins may serve as a biomarker for infectivity risk. For example, levels of ACE2 have also been shown to correlate with the risk of SARS-CoV-2 infection [13]. Thus, both ACE2 and TMPRSS2 may be putative targets for Covid-19 prevention.

Epidemiology can provide potential insight into therapeutic development by identifying factors that modulate disease in order to foster mechanistic studies and hence potential targets. While Covid-19 has recently emerged and thus limited long-term epidemiology, there is copious data on other coronaviruses since this viral family has been shown to be one of the more frequent causes of upper respiratory tract infections (URIs) [14]. Importantly, numerous studies support the role of exercise (physical activity) with a reduced risk of URIs [12,15,16]. The mechanisms through which moderate exercise may mediate URI reduction is likely pleiotropic encompassing both metabolic and immune changes. Recently, several lines of evidence indicate that cytokines elaborated by contracting muscle cells (termed myokines) may mediate some of the salutary impacts of exercise on health [17,18]. For instance, the anti-cancer effects of exercise have been shown to be mediated, at least partly, by myokines such as SPARC (colon cancer) and myostatin (breast cancer) [19]. One complicating factor that has stymied progress in the field is a large number of myokines with pleiotropic (~3000) and low circulation abundance. Therefore, cell culture models may provide an important modality for unravelling the role of myokines (versus other effects of exercise such as metabolic or immune stimulation) and serve as a platform for identifying particular molecules as potential future therapeutic agents.

The present study develops a novel approach to generate a system to replicate exercise in cell cultures. This *in-vitro* system successfully differentiated myoblasts (mouse cell line C2C12) into myotubule. The differentiated cells were contracted via electrical stimulation, followed by a concentration of conditioned media. The concentrated conditioned medium compares unstimulated myotubules to attest its efficiency and response to cells, which avoids some potential confounding approach. Therefore, the current study utilizes this methodology to assess the regulation of secreted myokines on ACE2 and TMPRSS2 expression in an immortalized human bronchial epithelium cell line. Hence, the study explores the therapeutic/preventive effect of *in vitro* produced myokines against COVID-19.

## Materials and methods

### Cell lines and differentiation

The immortalized normal human bronchial epithelial cell line 16HBE14o (Sigma-Aldrich, St. Louis, MO) and lung cancer cell line A549 (American Type Culture Collection, Manassas, VA) were used for *in-vitro* studies. The HBE cell line (human bronchial epithelium) was immortalized with SV40-based plasmid and is widely used in respiratory barrier studies [20] whereas A549 is a standard human adenocarcinoma cell line. 16HBE14o and A549 cells were cultured at 37°C, 5% CO_2_, in RPMI medium (Gibco, Invitrogen) supplemented with 10% fetal bovine serum and 100U/mL of penicillin and 100 μg/mL of streptomycin (Thermo Fisher Scientific, Waltham, MA). For myokine generation, the mouse myoblast cell lines C2C12 (American Type Culture Collection, Manassas, VA) was cultured at 37°C, 5% CO_2_, in Dulbeccos Modified Eagle Medium (DMEM) (supplemented with 10% fetal bovine serum and 100U/mL of penicillin and 100 μg/mL of streptomycin (Thermo Scientific, Waltham, MA). To differentiate C2C12 myoblasts into multinucleated myotubules, myoblasts were grown in 6-well plates to 90% confluency. Myoblasts were differentiated by treating cells with DMEM supplemented with 10% horse serum (Thermo Fisher Scientific, Waltham, MA), 5μg/mL insulin (Sigma Aldrich, St. Louis, MO), and 100U/mL of penicillin and 100 μg/mL of streptomycin in 37°C at 5% CO_2_. Cells were differentiated for 7–10 days, changing media every 1–2 days.

### Myokine generation and treatment

Myokines were generated using a protocol modification for C2C12 multinucleated myotubule contraction [21]. Optimized conditions were devised under the bright field microscope, C2C12s will have formed myotubules in >90% of the field of view (4x magnification). Myotubules appeared in long, continuous strands and organized in straight or curved patterns. Briefly, myotubules grown in 6 well plates were stimulated with a compatible carbon electrode using a low-frequency electric pulse stimulation (EPS) with bipolar pulses of 3.2 ms at 30 V and 1 Hz continuously using the Cell Pace EP (Ion Optix, Westwood, MA). Under electric stimulus >50% myotubules structures will display contraction (shortening and lengthening of tubules).

Cells were contracted in Krebs Ringer buffer (for stimulated myokine generation) or left in buffer without EPS (for unstimulated myokine control generation) for 8 h. Since these myotubules could secrete myokines without contraction (and there was sometimes low intermittent contraction noted without electrical stimulation), the comparator was the Krebs Ringer buffer from non-paced myotubules for the equivalent time frame incubated in these cells for 8 h. The Krebs buffer conditioned by the C2C12 cells was filtered using a 0.22 μm filter and centrifugated in Amicon Ultra centrifugal tubes at a molecular cut-off weight of 10 kDa (Sigma Aldrich, St. Louis, MO) to create a stimulated or unstimulated myokine batch. Approximately 10–12 mL of buffer was collected per 6-well plate of C2C12 cells in both stimulated in unstimulated conditions. These buffers were concentrated using 10 kDa Amicon ultra-centrifugal tubes, yielding 250 to 300 μL of a myokine fraction. For treatment of cells, the myokine fractions (stimulated or unstimulated control) were diluted to ~0.01% concentration in DMEM supplemented with 10% FBS.

### RNA isolation and quantitative RT-PCR

RNA from stimulated or unstimulated myokine-treated cells were isolated using Trizol (Thermo Fisher Scientific, Waltham, MA) and purified using the standard method. RNA quantification and purity were accessed using nanodrop (Thermo Fisher Scientific, Waltham, MA) by measuring 260/280 ratio. Complementary DNA (cDNA) synthesis was performed using the High-Capacity cDNA synthesis kit (Life Tech, Foster City, CA) using the manufacturer’s protocol. cDNA was used to perform ACE2, TMPRSS2, and GAPDH (endogenous control) TaqMan real-time gene expression assays (Thermo Fisher Scientific, Waltham, MA). Quantitative RT-PCR assays were performed with TaqMan fast advanced master mix in the Step One Plus thermocycler (Thermo Fisher Scientific, Waltham, MA). Relative gene expression was analysed through the 2^-ΔΔCT^ method [22].

### Enzyme-Linked Immunosorbant Assay (ELISA)

Protein was isolated from stimulated or unstimulated myokine treated 16HBE14o cells using NP-40 cell lysis buffer (Thermo Fisher Scientific, Waltham, MA) with the addition of 1x protease/phosphatase inhibitor. Protein was standardized using Bradford’s Quickstart protein assay (Bio-Rad Lab, Hercules, CA). ACE2 protein levels were assessed using the Human ACE2 ELISA kit (ABCAM, Cambridge, MA) following the manufacturer’s protocol. TMPRSS2 protein expression using the Human TMPRSS2 ELISA kit (Novus Biologicals, Centennial, CO). Absorbance was measured at 450nm using an Infinite M1000 plate reader (Tecan, Mannedorf, Switzerland). The minimum r^2^ values of the standard curve for valid assessment were deemed to be >0.95.

### Proteomics analysis of myokines

Unstimulated and stimulated myokine fractions were processed and analyzed by IQ Proteomics (Cambridge, MA) for mass spectroscopy [23,24]. IQ Proteomics has a next generation isobaric labeling strategy that utilizes a set of chemical reagents for tagging peptides with a stable isotope encoded barcode. The fundamental concept is that each tag within a multiplexed set using heavy isotopes barcode. Myokine fraction samples were processed and labeled with a 10-plex tadem mass tag using in house methods previously described [23,24]. TMT labeled peptides were analyzed for an SPS-MS3 based approach on an Orbitrap Fusion Lumos coupled to an Easy nLC-1200 HPLC (ThermoFisher Scientific, Waltham, MA). Peptides were identified via the SEQUEST algorithm and filtered to a 1% protein FDR and 1% peptide FDR through linear discriminant analysis utilizing a variety of parameters (XCorr, delta XCorr, PPM, and charge).

### Data analysis

Data analysis was performed to calculate means and standard error. Dixon’s Q-test was used for outlier value identification. Statistical significance was determined using a two-tailed student’s t-test using a threshold of p<0.05.

## Results

To investigate the effects of stimulated myokines as the exercise model, we chose the 16HBE14o cell line for our *in-vitro* target model. These cells were isolated from normal bronchial epithelium immortalized using SV-40 [25]. The bronchial epithelium is one of the important portals through which SARS-CoV-2 infects the lungs. This model has been utilized for studies with the SARS-CoV-2 infection, supporting the validity of this cell line as a suitable model for Covid-19 research [26,27]. One issue addressed while designing and performing these myokine experiments would be the biological variability in generated myokine batches. Although seeding and differentiation protocols have been standardized, these myoblasts are biological structures that are subject to some variability (degree and length of myotubule formation and differentiation, level of contraction with electric stimulation). There were modest batch-to-batch alterations in potency noted. To ensure scientific rigor in our studies, we utilized three distinct batches for these experiments. Each of the three experiments was conducted, in triplicate. Furthermore, to compare the results of each individual experiment, values were converted to the percentage of control.

### Myokines targets ACE2 mRNA 16HBE14o

**Fig 1:**
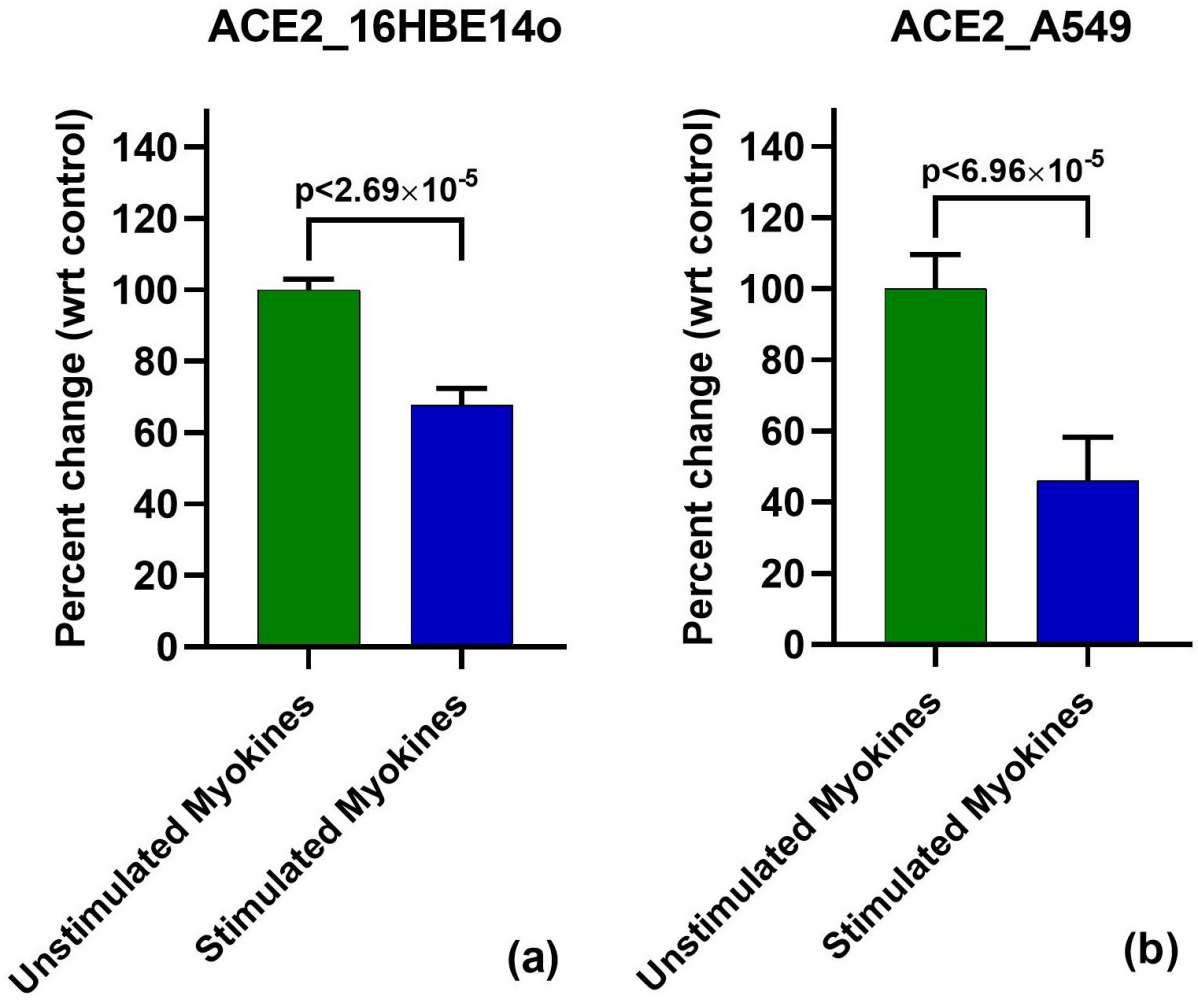
Downregulation of ACE2 mRNA with 48 h stimulated myokine treatment. **(a): ACE2 qPCR in 16HBE14o**. ACE2 mRNA was also shown to be downregulated with stimulated myokine treatment by 32.5%. **(b): ACE qPCR in A549**. Stimulated myokine treatment resulted 63.9% downregulation of ACE2 gene expression.

To determine the performance of myokines against SARS-CoV-2 entry in the host cell, we investigated the mRNA levels of ACE2 in immortalized bronchial epithelial cells. In 16HBE14o cells, 48 hour treatment of stimulated myokine treatment showed minimal batch to batch variability and greater downregulation of ACE2 mRNA versus earlier or later timepoints (24 and 72 hours respectively). Each experiment showed a modest reduction (28.0%-34.8%) in ACE2 with stimulated vs. unstimulated myokine treatment. Taken together, the stimulated myokine treatment showed a 32.5% reduction of ACE2 expression, with statistically high significance (p<2.69x10^-5^) (Fig 1a).

### Corroboration of ACE2 decrease by exercise-induced myokines in A549 cells

For additional scientific rigor to ensure that our observed effects of stimulated myokines on ACE2 is not cell line-specific but more generalizable, we also used the human non-small cell lung cancer cell line A549 to validate our findings. Although A549 cells are transformed, they have served as models of alveolar Type II pulmonary epithelium studies [28,29]. For these experiments, A549 was treated with different batches of stimulated or unstimulated myokines for 48 h in three independent experiments to assess ACE2 mRNA levels.

Through real time PCR we determined the effects of myokines on ACE2 transcript in A549 cells by observing a marked downregulation with stimulated myokine treatment. This exhibited a much more striking effect as compared to stimulated myokine treatment in non-transformed 16HBE14o cells. We observed between 52.3–81.1% reduction in ACE2 mRNA in the three independent experiments. When combined, our experiments displayed a 62.6% downregulation in ACE2 mRNA in A549 cells (p<6.96x10^-5^) (Fig 1b).

### Correlation of ACE2 protein levels

Given the reduction in mRNA, we wanted to confirm that this effect in 16HBE14o cells progressed on a translational level. 16HBE14o cells was an attractive model as it possessed a substantial basal level of ACE2 as compared to A549 cells. Real time PCR revealed A549 cells had 94.2% lower expression of ACE2 mRNA (p<2x10^-8^) as compared to 16HBE14o.

**Fig 2:**
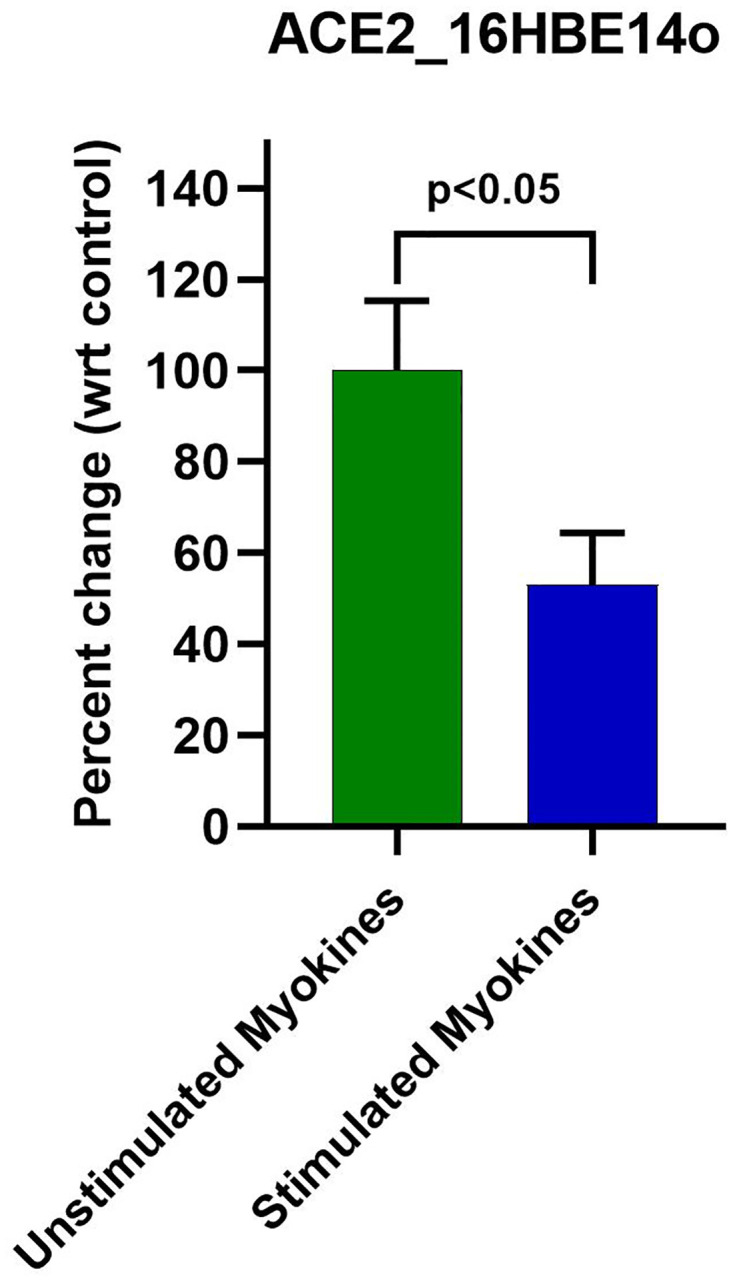
48 h stimulated myokine treatment downregulated ACE2 protein levels in 16HBE14o. Anti-ACE2 ELISA revealed a ~53.0% downregulation of ACE2 protein with stimulated myokine treatment.

Given the relatively higher levels of ACE2 mRNA we incorporated an anti-ACE2 ELISA for further investigation; moreover this substantiates the relevance of 16HBE14o for these studies. Three independent ELISAs were performed using different batch treatments of either stimulated or production-matched unstimulated control. Post 48h myokine treatment, ACE2 protein was reduced between 42.0–49.4%, consistent with ACE2 mRNA downregulation. Together, these experiments displayed a 46.9% reduction in ACE protein (p<0.05) (Fig 2).

### TMPRSS2 mRNA levels in 16HBE14o and A549

**Fig 3:**
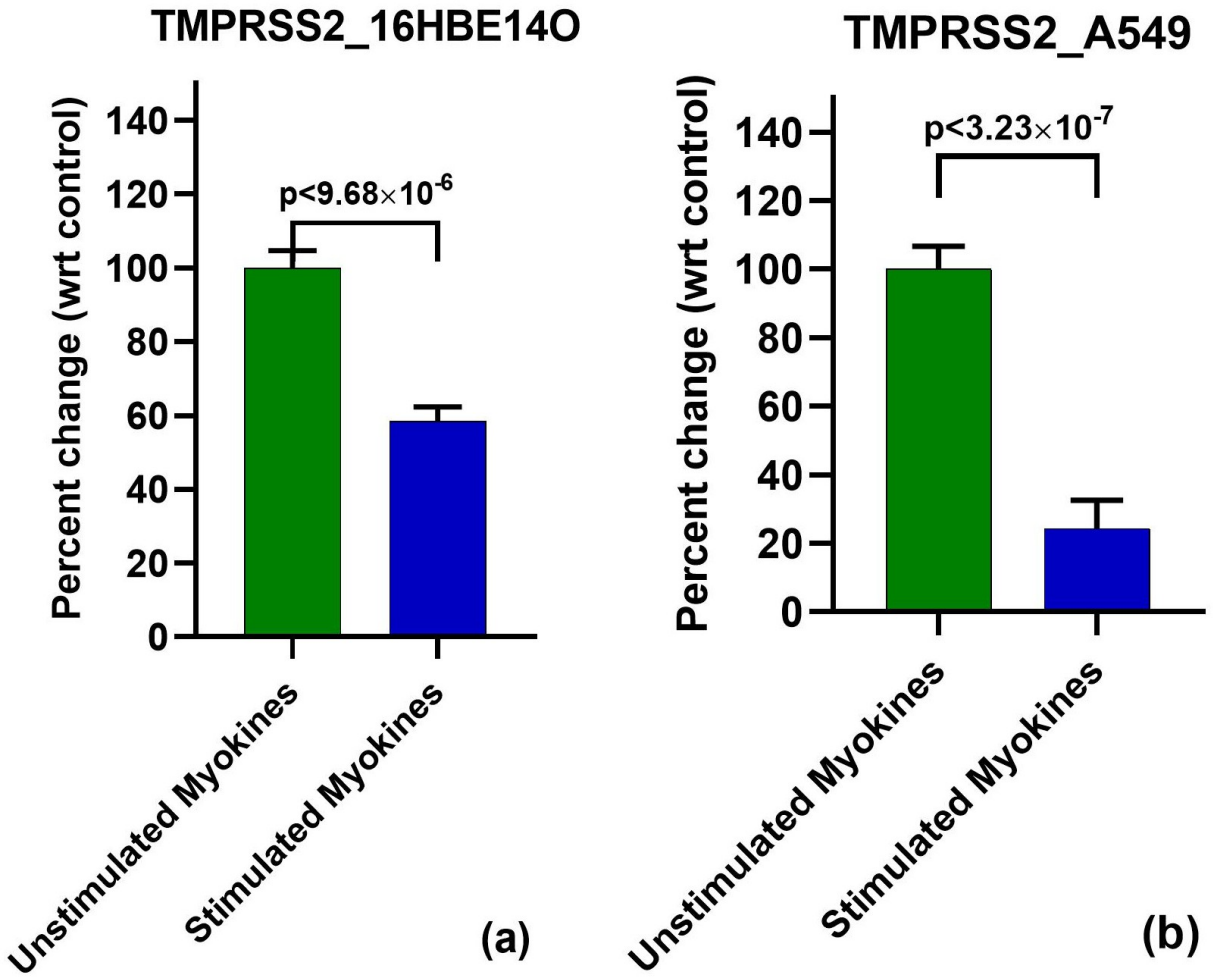
Downregulation of TMPRSS2 mRNA with 48 h stimulated myokine treatment. **(a): TMPRSS2 qPCR in 16HBE14o**. Real time pcr revealed a 41% reduction of TMPRSS2 mRNA with stimulated myokines. **(b): TMPRSS2 qPCR in A549**. Stimulated myokine treatment reduced TMPRSS2 gene expression by 75.7%.

We further examined the potential significance of exercise-induced myokines in targeting SARS-CoV-2 entry by exploring TMPRSS2 mRNA expression in myokine-treated 16HBE14o cells. Similar to the effect on ACE2, we observed a modest reduction of TMPRSS2 transcript at 48 h stimulated myokine treatment compared to unstimulated myokines treatment, further supporting the potential effects of stimulated myokines against of SARS-CoV-2 entry. We performed three independent experiments (in which a different myokine batch per experiment was used), which displayed a 30.4–47.3% reduction of TMPRSS2 mRNA with stimulated myokine treatment. When combining all three experiments, we showed downregulation of TMPRSS2 by exercise-induced myokines (as compared to non-contracted myokines) by statistically convincing 41.5% (p<4.57x10^-5^) (Fig 3a).

TMPRSS2 mRNA levels in stimulated myokine treated A549 cells were also investigated to determine if this treatment exhibits comparable effects compared to 16HBE14o cells. In this regard, we observed a potent effect on the TMPRSS2 transcript, where our replicate experiments showed a 61.9–86.0% downregulation with stimulated vs. unstimulated myokine treatment. Together, these experiments revealed a 75.7% reduction of TMPRSS2 mRNA in A549 (p = 3.23x10^-7^) (Fig 3b), consistent with the effects of stimulated myokines on 16HBE14o cells, albeit at a higher efficacy.

### Validation of TMPRSS2 protein levels

When assessing the baseline expression of TMPRSS2, A549 cells exhibit a ~97.0% lower mRNA expression when comparing their threshold cycle (CT) to 16HBE14o cells (5.93x10^-10^).

**Fig 4:**
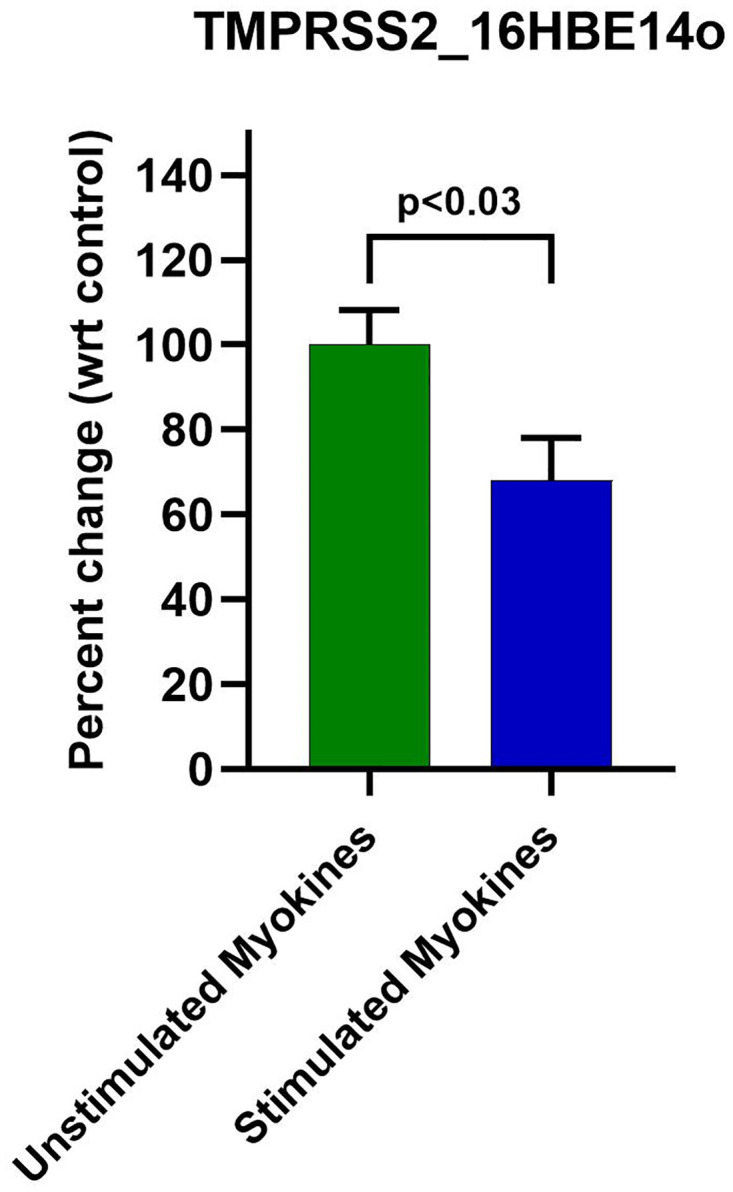
48 h stimulated myokine treatment downregulated TMPRSS2 protein levels in 16HBE14o. Anti-TMPRSS2 ELISA revealed a ~32% reduction of TMPRSS2 protein with stimulated myokine treatment.

Given the downregulation of TMPRSS2 mRNA with stimulated myokine treatment, we wanted to investigate further whether stimulated myokines could downregulate TMPRSS2 protein levels in 16HBE14o cells through TMPRSS2 ELISA. Three independent experiments with different batches of myokines displayed a modest reduction of TMPRSS2 protein by 13.2–66.1%. Taken together, stimulated myokine treatment displayed a modest ~32.0% reduction in TMPRSS2 protein (p<0.03) (Fig 4).

### Pilot proteomic data

**Fig 5:**
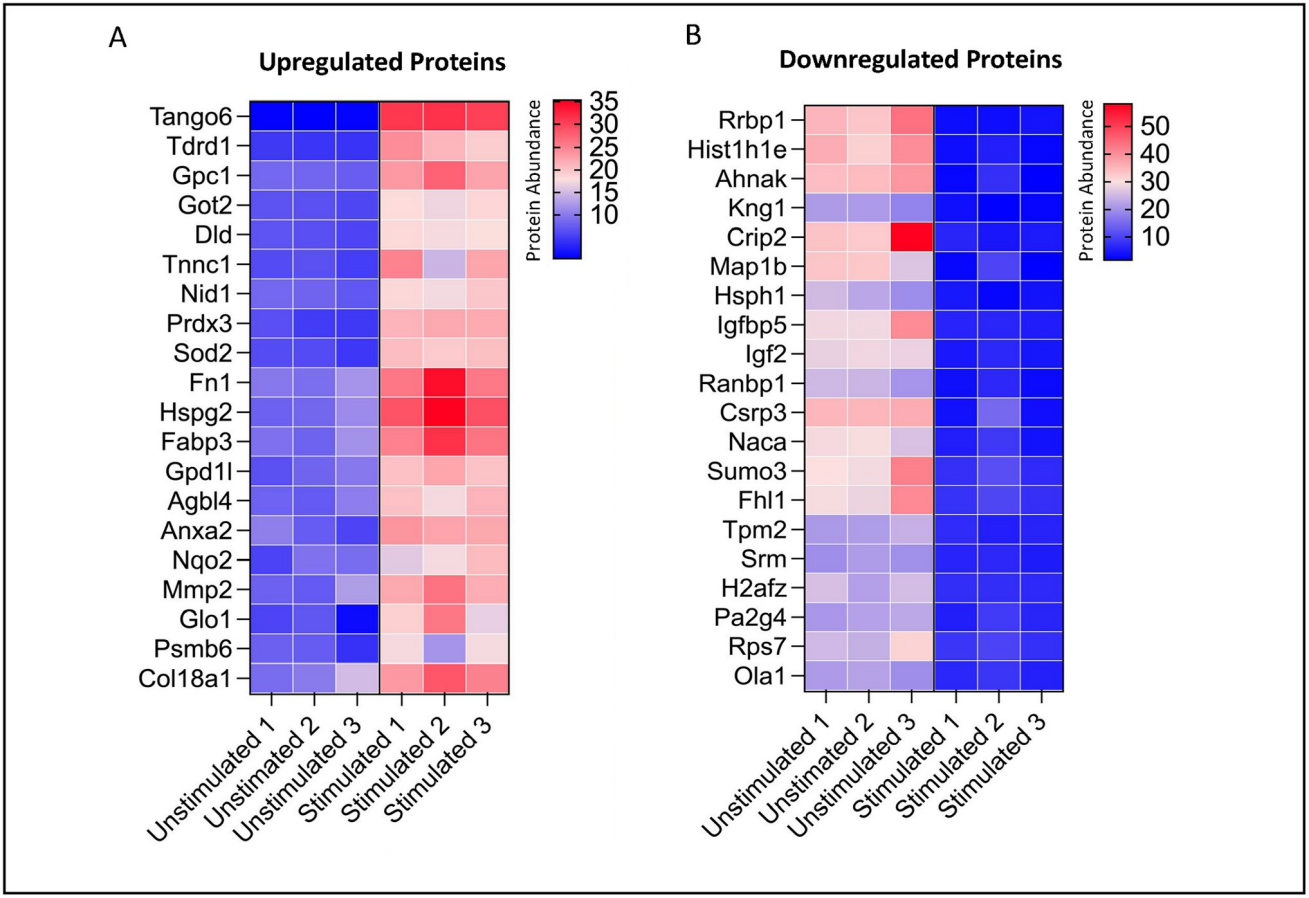
Heat Map of Top 20 upregulated and downregulated proteins in myokine fractions. Proteomic characterization through Tandem Mass Tag Label Mass Spectroscopy revealed marked changes in protein abundance between stimulated and unstimulated myokine fraction. A. Top 20 upregulated proteins in stimulated myokine fraction possessed marked inductions as compared unstimulated control. B. Top 20 downregulated proteins in stimulated myokine fraction also displayed the ability of the stimulated myokine fraction to markedly downregulate protein abundance as compared to unstimulated myokine fraction.

We performed proteomic analysis on conditioned media from myotubules from C2C12s that were either electrically stimulated/contracted for 8 hours (n = 3) or left unstimulated as controls (n = 3). Our proteomics quantified 6,040 peptides which had 3,156 unique peptides with 704 quantified proteins. There was excellent discrimination by principal component analysis. We present the top 20 upregulated and downregulated proteins (abundance changed by at least 50%) (Fig 5).

## Discussion

We provide the first experimental evidence that exercise may suppress SARS-CoV-2 infection. For these studies, we employed modifications of the cell culture system to generate exercise-induced myokines. Our target was the SARS-CoV-2 receptor, ACE 2, with an immortalized human bronchial cell line (6HBE14o) as a modality of mirroring human respiratory infection [26,27]. We showed that exercise-induced myokines caused a marked decrease in ACE2 protein when compared to myokines derived from non-contracting myotubules. The difference was more accentuated in mRNA, suggesting that the impact was at the transcriptional level.

Furthermore, the greater magnitude of mRNA in these relatively short-term experiments would suggest that longer experiments might provide more substantial effects. For additional rigor, we reproduced the work in a transformed lung cell line (A549). Moreover, this was paralleled by data from TMPRSS2. Finally, it was also observed that TMPRSS2, which also impacts the ACE2 interaction with spike protein of SARS-CoV-2, showed significant downregulation in both 16HBE14o and A549 cell lines.

ACE2 is the epithelial receptor for the SARS-CoV-2 Spike protein [8]. Teleologically, mucosal ACE2 levels should correlate with the propensity for SARS-CoV-2 infection. This is corroborated by animal studies where transgenic mice expressing human ACE2 receptors were compared with wildtype mice challenged with SARS-CoV-2 [30,31]. Recent findings by Bao *et al*. [32] reported that mice overexpressing hACE2 were infected with SARS-CoV-2 and developed the pulmonary disease, mortality, and viremia compared to wildtype mice. These results demonstrate SARS-CoV-2 pathogenicity in mice which testifies Koch’s postulates [33]. Further, regarding clinical data, Bunyavanich and colleagues [34] showed that infants and children who are generally less susceptible to infection had a low nasal ACE2 level, which increased with age and was proportional to high infection risk [2]. Wallentin et al. [35] also showed that known Covid-19 risk factors such as the male gender, cardiovascular disease, and diabetes were associated with higher serum ACE2 levels.

Thus, our studies provide cell culture support for exercise to reduce the risk of SARS-CoV-2 infection. While there is no specific epidemiological evidence to support the exercise and Covid-19 link, we could extrapolate from viral URIs, which are commonly caused by coronaviruses and rhinoviruses, parainfluenza, etc. In this regard, there is a wealth of observational studies showing the benefit of exercise in decreasing URIs. For instance, Niemann and colleagues [36] followed 1,002 patients for 12 weeks over fall and winter. Active subjects (≥5 days/week aerobic exercise) had a 43% lower risk of URI versus those who were sedentary (≤1 day/week of aerobic exercise) the URI rate. Additionally, Fondell and colleagues [37] followed 1509 Swedish people over 4 months with every three weeks high levels of physical activity assessment and self-reported URI incidence. When compared to those with lower activity (<45 MET·h·d), those with higher activity (≥ 55 MET·h d) was associated with an URI incidence rate ratio of 0.82, 95% (CI) = 0.69–0.98). Similar findings have been demonstrated in randomized, controlled trial such as one performed by Barrett et al. [12] in an 8 week study with 84 subjects in moderate exercise arm and 82 in the control group. A multivariate regression model suggested that exercise reduced global severity for URI (p = 0.042) [12]. A recent Cochrane analysis was hampered by a small study size, heterogeneous population, and risk of bias thus, they were unable to definitely demonstrate that moderate exercise reduced URI incidence [16]. However, they did note that exercise resulted in a statistically significant reduction severity of URI symptoms. Moreover, a meta-analysis concluded that physical activity reduced 31% community-acquired infectious and 37% decrease in mortality rate [38]. Further supporting the role of exercise is immune studies as demonstrated by a significant CD4 cell count and salivary immunoglobulin IgA concentration. In this regard, patients with an adjunct physical activity program manifested a higher antibody concentration after vaccination [38]. While epidemiological evidence is compelling, there are nuances with the curve of exercise dosage and URI being non-linneear but actually J-shaped curve [39]. Novas and colleagues followed 35 girls over a 12 week period of time with activity diaries [40]. They noted that moderately active girls (quartile 3) had a lower risk of URI symptomatology than those in the lowest quartile. On the other hand, girls with the highest total daily energy expenditure (top quartile) showed more URI symptomatology [40]. Intriguingly, mucosal ACE2 levels may also indicate a non-monotonic relationship with the severity of SARS-CoV-2 infection, due to the fact that ACE2 is not only the receptor of SARS-CoV-2, but ACE2 also suppresses the exuberant immune response that can trigger acute respiratory distress syndrome (ARDs), a major reason for Covid-19 fatalities. Studies of transgenic mice have shown that knocking down the ACE2 drastically suppressed SARS-CoV-2 infections [35]. However, acid-induced lung injury models have shown a protective role of ACE2 from acute lung injury induced by acid aspiration or sepsis with greater severity inflammation/ARDS and mortality [41]. These clinical and pre-clinical studies attest to the key role of ACE2 in the development and pathogenesis of acute lung injury. Therefore, this provides a potential caveat of relying solely on ACE2 as a marker of myokine efficacy.

For the further support of the present study, we also considered TMPRSS-2 as a potential marker. As noted, TMPRSS2, a serine protease, cleaves the SARS-CoV-2 spike protein to provide the “lock and key” interaction with the epithelial cell ACE2 [42]. In many ways, TMPRSS2 is a promising inhibitor, which should be directly proportional to the suppression of SARS-CoV-2 infection [43–45]. Indeed, TMPRSS2 protease agents like camostat mesylate have also been suggested as a potential antiviral drug against SARS-CoV-2 [46,47]. In our studies, we noted that exercise-induced myokines downregulated the TMPRSS2 in fashion that mirrored ACE2 fashion (~50%) and appeared to be rigorous (reproducible at mRNA and protein level and corroboration of expression data in replicated in second cell line).

There are a variety of strengths of this study. Our study focused on supporting the concept of exercise through myokines. The *in vitro* model has significant advantages over clinical or animal studies in that the myokine concentration in the blood is low when compared to the myriad of other circulating proteins. Furthermore, a cell culture system may be useful given the complexity of the myokinome (~3000 molecules) for future identify of specific molecule(s) [17]. In addition, second cell line not only provides scientific rigor but supports generalizability (malignant and immortalized cell lines). The current study was designed simply to demonstrate that exercise induced myokines may alter ACE2 and TMPRSS-2 expression. However, future studies will focus on identifying particular myokine(s) with synergistic impact on ACE2/TMPRSS2 downregulation and avoiding myokines with antagonistic effects. The studies include the concentration of myokines >10kDa, which provides some selectivity, although in the conditioned media there are likely to be a multitude of molecules [17,18,21]. With regards to the target tissue, we posit that cell surface receptor activation appears to be the most likely mechanism although given data suggests that myokines can be released in exosomes and may provide a modality for direct intercellular targeting [48]. Mechanistically, epigenetic research has revealed that methylation and post-translational histone modification can impact ACE2 expression providing potential patters of viral replication and infection [49,50]. However, further studies need to expand upon our current proof of principle that exercise induced myokines can suppress important proteins needed for SARS-CoV-2 infections. It is intriguing to speculate that since total ACE2 levels are decreased, this approach could be robust despite Spike protein mutations characteristic of Covid-19 variants.

While our data is robust, there are a several limitations that need to be acknowledged. First, while the immortalized bronchial cell line appears appropriate, the extrapolation of cell culture studies to animals and humans can be fraught with difficulty. The use of ACE2 and TMPRSS2 as a surrogate marker of SARS-CoV-2 infectivity risk seems reasonable but clearly requires confirmation about reduced infectivity *in vivo*. Our data is derived from a collection of all myokines elaborated by C2C12. It is conceivable that there may be different myokine(s) involved in suppression or ACE2 and TMPRSS2. On the other hand, seeing an effect with the mixture of myokine (mimicking the *in vivo* condition) might mean that the purified myokine(s) may have a bigger effect since the mixture may potentially dilute out the effects of the active myokines or even have antagonistic effects. Moreover, the dosage of the putative active myokines has not been optimized and thus it is plausible that the effect seen may potentially be improved. Finally, we reiterate that these studies are preliminary and clearly require corroboration in future animal models and eventually human trials.

## Conclusion

In conclusion, we demonstrate biological data that supports the protective effect of exercise against Covid-19. This proof of principle study further strengthens the role of myokines as potential mediators for the beneficial effects of exercise. From a therapeutic perspective, identifying the myokine(s) involved may allow novel approaches against SARS-CoV-2 and similar viruses although the current cell culture-based work is very preliminary.

## Supporting information

S1 File (XLSX)
